# Interventricular septum hematoma during cineventriculography

**DOI:** 10.1186/1476-7120-6-4

**Published:** 2008-01-16

**Authors:** Andrea Grohmann, Thomas Elgeti, Stephan Eddicks, Fabian Knebel, Wolfgang Rutsch, Christoph Melzer, Gert Baumann, Adrian C Borges

**Affiliations:** 1 Department of Cardiology and Angiology, Charite Campus Mitte, Universitätsmedizin Berlin, Berlin, Germany; 2 Department of Radiology, Charite Campus Mitte, Universitatsmedizin Berlin, Berlin, Germany

## Abstract

**Background:**

Intraseptal hematoma and subsequent myocardial infarction due to accidental contrast agent deposition complicating diagnostic cineventriculography is a previously undescribed complication of angiography.

**Case presentation:**

A 61 year old man was admitted at intensive care unit because of unstable angina pectoris 1 hour after coronary angiography. Transthoracic contrast echocardiography showed a non-perfused area in the middle of interventricular septum with an increase of thickening up to 26 mm. Review of cineventriculography revealed contrast enhancement in the interventricular septum after contrast medium injection and a dislocation of the pigtail catheter tip. Follow up by echocardiography and MRI showed, that intramural hematoma has resolved after 6 weeks. After 8 weeks successful stent implantation in LAD was performed and after 6 month the patient had a normal LV-function without ischemic signs or septal thickening demonstrated by stressechocardiography.

**Conclusion:**

A safe and mobile position of the pigtail catheter during ventriculography in the middle of the LV cavity should be ensured to avoid this potentially life-threatening complication. For assessment and absolute measurement of intramural hematoma contrast-enhanced echocardiography is more feasible than MRI and makes interchangeable results.

## Background

This case illustrates the first description of an accidental contrast agent deposition into the interventricular septum complicating cineventriculography in a patient with suspected coronary artery disease. Intramural hematoma developed subsequently-leading to myocardial infarction.

## Case presentation

A 61 year old patient with sudden onset of typical angina pectoris, sweat and a sinus bradycardia of 40 beats per minute was admitted at intensive care unit. It was known, that he had an elective coronary angiography at the same day because of suspected coronary artery disease with typical chest pain and exercise induced dyspnoea as well as paroxysmal atrial fibrillation. His baseline treatment was for arterial hypertension metoprolol (150 mg/d) and ramipril (2,5 mg/d), for atrial fibrillation dalteparin (3 × 5.000 IU/d) and for expected angioplasty aspirin (100 mg/d) and loading dose of clopidogrel (300 mg at the first day). The coronary angiography report described a severe proximal type B2 stenosis and a medial type B stenosis of the left anterior descending artery (LAD). Intended angioplasty of the LAD and implantation of a special stent was planned for the next day.

**Figure 1:**
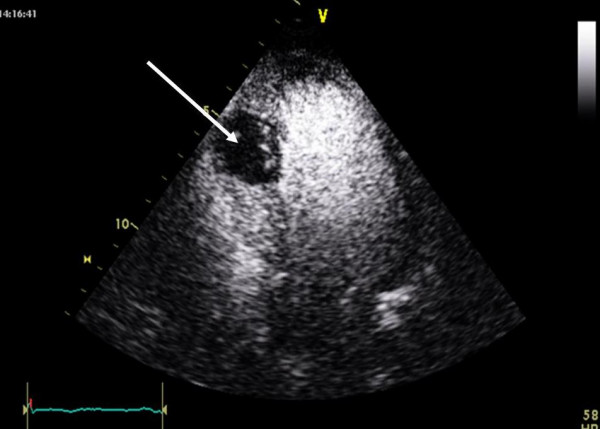
The dark color shows the intraventricular septum thickening (26 mm) and loss of perfusion in the medial septal segment (white arrowhead) demonstrated with low mechanical index contrast echocardiography.

**Figure 2:**
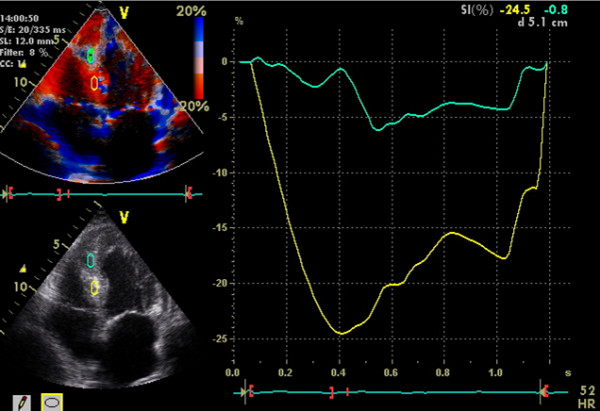
Normal strain of basal intraventricular septum with 25% (yellow curve) and pathologic strain of middle intraventricular septum with 6% (green curve).

At intensive care unit an ECG was performed because of unstable angina pectoris one hour after finishing angiography but without signs of acute myocardial ischemia. Transthoracic echocardiography (ViVid 7, GE Vingmed, Norton, Norway; 7,5 MHz ; very low non destructive transmit power technique, MI 0,12, non triggered mode; contrast agent Sonovue as bolus of 0,1 ml) demonstrated an increase of septum thickness from 13 mm to 26 mm. Septal strain (6%) as well as strain rate (0,45/s) showed an non perfused areal of 2,3 × 3,3 cm in the middle of interventricular septum. (Figure 1 and 2).

**Figure 3:**
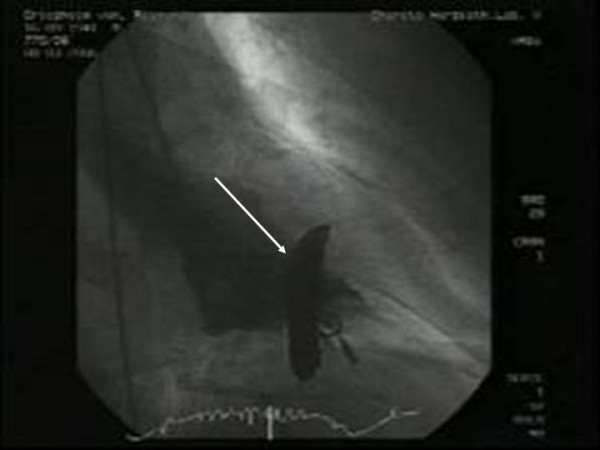
Arrow indicates the contrast medium in the interventricular septum.

These echocardiographic and clinical findings were in accordance with an acute non-ST-segment elevation infarction of the interventricular septum in connection with angiography. Cineventriculography was reviewed again and revealed a contrast enhancement in the interventricular septum after conventional automated high flow contrast medium injection. Tip of pigtail catheter was dislocated in between the trabeculae of the interventricular septum (Figure 3).

CK max value of 1082 U/l and Troponin I I value of 80 ng/ml were observed during the same day. The following day ECG showed ST-segment elevation in V1 (+0.15 mV) and V2 (+0.25 mV) indicating myocardial infarction. No ventricular arrhythmias or atrio-ventricular block caused by the septal infarction were observed. 2 days after contrast agent deposition into interventricular septum CK values decreased dewithin normal range. 2 weeks later, transthoracic echocardiography revealed a maximum of septum thickness with 28 mm, a non-perfused area of 2.6 × 4.7 cm with a strain of -4% and a strain rate of 0,45/s as well as an akinesia in the middle septum.

**Figure 4:**
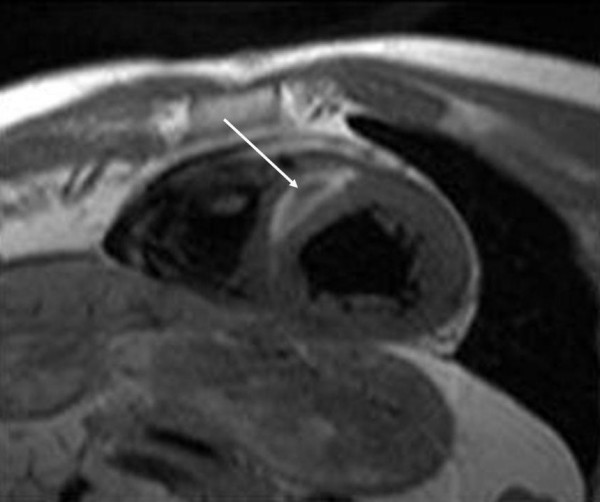
T1 weighted image in the short cardiac axis. Bright signal within the myocardium of the intraventricular septum shows intramyocardial hematoma (white arrow).

**Figure 5:**
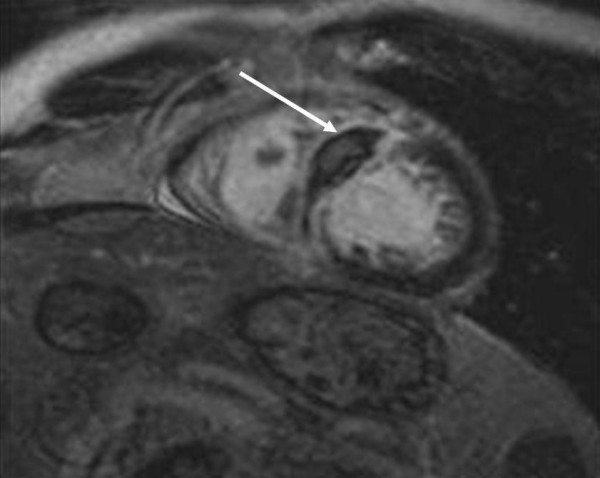
T1 weighted image, after application of 0,2 mmol/kg body weight Gadolinium-DTPA with a preparation pulse to "null" normal myocardium. A bright signal, surrounding the hematoma, indicating myocardial fibrosis can easily be depicted (late gadolinium enhancement, arrowhead).

By magnetic resonance tomography an intramural hematoma was demonstrated 3 weeks after contrast medium injection into interventricular septum (Figure 4 and 5).

**Figure 6:**
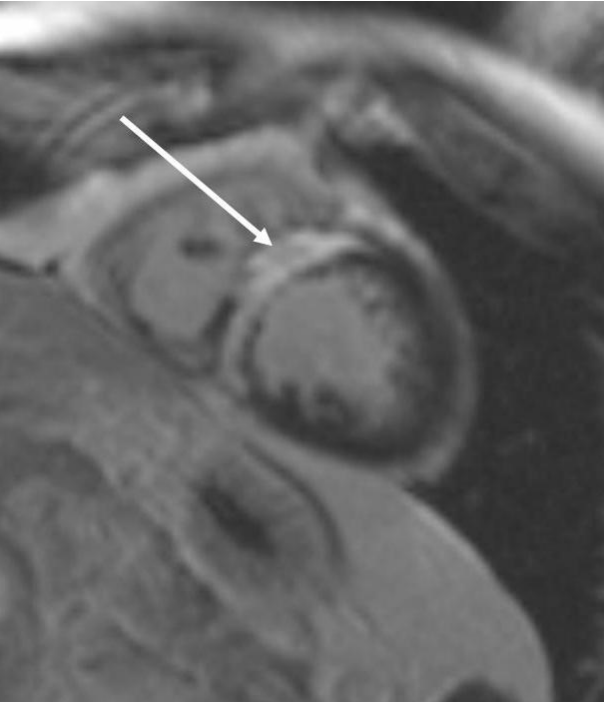
MR-follow-up examination 8 weeks after contrast agent injection (same technique as described under Figure E): Transmural late gadolinium enhancement anteroseptal, transmural myocardial fibrosis (white arrow), no hematoma.

6 weeks after contrast agent injection the baseline septum thickness (13 mm) was restored, the non-perfused region was reduced to 0.9 × 1 cm in mid septum with a strain of -7% and a width of 9 mm. 8 weeks after accidental intraseptal contrast agent injection patient underwent an elective angioplasty with successful implantation of two bare metal stents into LAD. At that point a MR-follow-up was performed (Figure 6).

**Figure 7:**
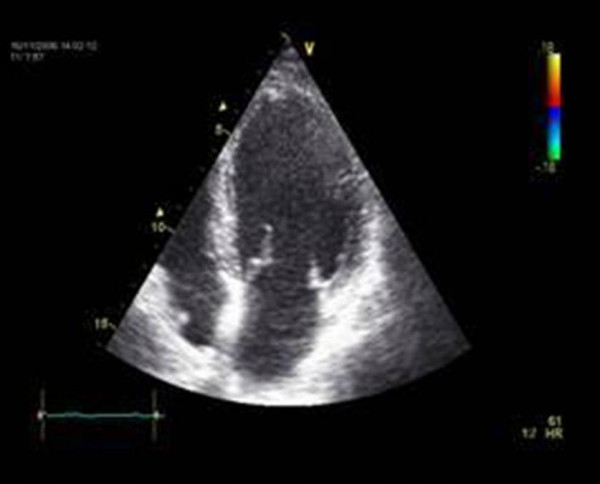
Enddiastolic 4 chamber view of left ventricle.

**Figure 8:**
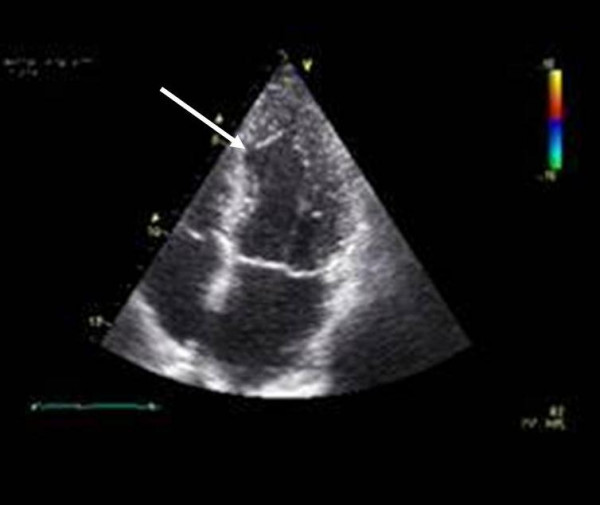
Endsystolic 4 chamber view of left ventricle: midseptal akinesia and wall thinning (white arrowhead).

The final pharmacological dobutamin-stressechocardiography 8 month after complicated cineventriculography and 6 month after successfully implanted LAD stents revealed a negative viability (contractile reserve) midseptal but a normal global systolic left ventricular function without regional stress induced myocardial ischemia. (Figure 7 and 8).

## Discussion and conclusion

Intramyocardial hemorrhage is a rare but known complication of myocardial infarction, percutaneous coronary artery intervention or angiography [1-7]. Intraseptal hematoma after ventricular septal defect closure is described in infants and requires surgically treatment [8,9]. An interventricular septum hematoma and myocardial infarction due to accidental contrast agent deposition complicating diagnostic cineventriculography is a previously undescribed complication of angiography. Intramural hematoma can be detected by contrast echocardiography [10] and magnetic resonance imaging [11]. Our findings confirm interchangeable results of contrast echocardiography and MRI regarding to absolute measurements and reproducibility [10]. Furthermore, in our case the assessment of myocardial perfusion and scar by contrast echocardiography is as good as that of MRI.

The possible reason for this complication seems to be the in correct position of pigtail catheter followed by high pressure injection of contrast medium intramuraly. In addition complex strategy of antiplatelet- and anti Xa-medication may have forwarded rupture of vasa vasorum.

## Conclusion

In conclusion, a safe and mobile position of the pigtail catheter during ventriculography in the middle of the LV cavity should be ensured to avoid this potentially life-threatening complication. For assessment and measurement of intramural hematoma contrast-enhanced echocardiography is more feasible than MRI and makes interchangeable results.

## Authors' contributions

GA: carried out the stressechocardiography, participated in the initial contrast-enhanced-, conventional and tissue-doppler echocardiography, participated in the sequence alignment and drafted the manuscript

ET: carried out the follow-up MRI and drafted the manuscript concerning MRI-technique.

ES: carried out the first MRI.

KF and BM: participated in the follow-up echocardiographic examinations and made comments to the manuscript.

MC: participated in the sequence alignment.

RW: did the off-line analysis and interpretation of cineventriculography.

BG: chief of the clinic, supervision.

BAC: performed initial echocardiography and is supervisitor of all echographic examinations, participated in sequence alignment, made final comments to the manuscript.
